# Loneliness and all-cause mortality in the United States

**DOI:** 10.1371/journal.pone.0354773

**Published:** 2026-09-25

**Authors:** Oluwasegun Akinyemi, Oluebubechukwu Eze, Mojisola Fasokun, Ifeoma Mba-Madubuike, Temitayo Ogundipe, Delia Singleton, Ayomide Ogunsakin, Samar Khalil, Kaelyn Gordon, Miriam Michael, Kakra Hughes, Temitope Ogundare

**Affiliations:** 1 The Clive O. Callender Outcomes Research Center, Department of Surgery, Howard University College of Medicine, Washington, District of Columbia, United States of America; 2 Department of Epidemiology, University of Alabama at Birmingham, Alabama, United States of America; 3 Department of Hospital Medicine, Sentara Williamsburg Regional Medical Center, Williamsburg, Virginia, United States of America; 4 Department of Internal Medicine, Howard University College of Medicine, Washington, District of Columbia, United States of America; 5 Department of Surgery, Howard University College of Medicine, Washington, District of Columbia, United States of America; 6 Department of Psychiatry, New York Presbyterian Columbia University Medical Center & Columbia University Vagelos College of Physicians and Surgeons, New York, New York, United States of America; Stamford Health System: Stamford Hospital, UNITED STATES OF AMERICA

## Abstract

**Importance:**

Loneliness affects more than half of US adults and has been declared a public health epidemic, yet whether clinically documented loneliness independently predicts mortality across diverse populations remains unclear.

**Objective:**

To examine the association between clinically documented loneliness and all-cause mortality at 1, 3, and 5 years, overall and by sex, race and ethnicity, and age.

**Methods:**

We conducted a propensity score–matched retrospective cohort study using the TriNetX US Collaborative Network, comprising electronic health record data from 65 health care organizations. Adults aged 18–90 years with at least one clinical encounter between January 1, 2016, and December 31, 2022, were eligible. Patients with a clinically documented diagnosis of loneliness were matched 1:1 (n = 28,944 per group) on age, sex, race and ethnicity, and 12 comorbidity domains to comparison patients with outpatient engagement and no loneliness diagnosis. The primary outcome was all-cause mortality at 1, 3, and 5 years. Between-group differences were assessed using risk differences, Kaplan-Meier survival analysis, and Cox proportional hazards models, overall and across prespecified subgroups.

**Results:**

Among 57,888 matched adults (mean age, 55.4 years; 62.9% female), loneliness was associated with increased mortality at every horizon: 1-year hazard ratio (HR), 1.52 (95% CI, 1.40–1.66); 3-year HR, 1.35 (95% CI, 1.27–1.43); and 5-year HR, 1.29 (95% CI, 1.22–1.35). Absolute survival gaps widened from 1.57 to 2.82 percentage points. The largest 1-year relative excess occurred among male patients (HR, 1.85) and Hispanic patients (risk ratio, 2.33). Black patients had the greatest 3-year absolute gap (2.49 percentage points). Working-age adults showed sustained excess hazard across all windows (HR, 1.34–1.42), whereas older adults had the largest 1-year absolute gap (2.28 percentage points) with convergence by 5 years.

**Conclusion:**

Clinically documented loneliness was independently associated with increased all-cause mortality at 1, 3, and 5 years, with the burden varying by sex, race and ethnicity, and age. These findings identify loneliness as a measurable, independent mortality risk marker and suggest that routine screening and targeted psychosocial intervention warrant prospective evaluation.

## Introduction

Loneliness, the subjective distress arising from a perceived deficit in the quality or quantity of social connection, has emerged as a defining public health challenge of the twenty-first century [1–3]. In 2023, the US Surgeon General declared loneliness a public health epidemic, noting that more than half of US adults report measurable loneliness and that its prevalence has nearly doubled over two decades [4]. It disproportionately affects older adults, racial and ethnic minorities, economically precarious working-age adults, and individuals with chronic illness populations already bearing the greatest burden of preventable morbidity and mortality in the United States [5–9]. The costs are substantial: loneliness is associated with greater healthcare use, reduced productivity, and premature mortality, and related social isolation among older adults accounts for approximately 6.7 billion dollars in excess annual Medicare spending [10–12].

The biological pathways linking loneliness to premature death are increasingly well-characterized [1,4,13,14]. Loneliness activates hypothalamic-pituitary-adrenal stress pathways, elevating cortisol and catecholamines that drive systemic inflammation, immune dysregulation, and endothelial dysfunction [13,15–17]. Loneliness and social isolation have been linked to incident cardiovascular disease, stroke, dementia, and increased all‑cause mortality, with meta‑analytic evidence linking both to a 25–30% higher risk of premature death, comparable to major risk factors such as smoking and obesity [1,18–21]. Despite this evidence, the clinical codification of loneliness as a diagnosable, actionable condition has lagged, and its independent contribution to mortality adjusted for the comorbidities it both predates and precipitates remains incompletely quantified [22,23].

Existing studies of loneliness and mortality face methodological limitations that constrain translational utility [18,24]. Most rely on self-reported scales administered in surveys, which do not reflect the clinically documented loneliness captured in health systems and cannot be operationalized into screening protocols or billing codes [25]. Prospective cohorts have largely been limited to older adult or single-sex populations, with follow-up rarely beyond three years, leaving the trajectory of excess mortality across 1-, 3-, and 5-year horizons largely undefined [24,26]. Critically, no large-scale analysis has examined whether the loneliness–mortality association differs in magnitude, temporal structure, or proportional hazard behavior across sex, racial and ethnic, and age-stratified subpopulations the comparisons most relevant to targeted intervention and health equity policy [18].

To address these gaps, we leveraged the TriNetX US Collaborative Network a federated real-world electronic health record platform spanning 65 health care organizations and millions of patient records to conduct a propensity score–matched cohort study of clinically documented loneliness and all-cause mortality [27,28]. This design offers several advantages: exposure is operationalized through ICD-10-CM codes, enabling direct linkage to clinical workflows; 1:1 matching on age, sex, race and ethnicity, and 12 comorbidity domains controls the confounding burden inherent to loneliness research; and the network’s scale enables adequately powered subgroup analyses across sex, racial and ethnic groups, and age strata not previously reported simultaneously [27–29].

In this study, we examined the association between clinically documented loneliness and all-cause mortality at 1, 3, and 5 years in propensity score–matched cohorts of US adults, and assessed whether it differed by sex, race and ethnicity, and age group. Our findings provide a longitudinal, clinically grounded evidence base for integrating loneliness screening into preventive health frameworks and informing equity-centered responses to the US loneliness epidemic.

## Materials and methods

### Study designstudy and data source

This propensity score–matched retrospective cohort study used de-identified electronic health record (EHR) data from the TriNetX US Collaborative Network, a federated platform comprising 65 health care organizations (HCOs) across the United States [27,30]. Data included diagnoses, procedures, medications, laboratory values, and mortality status [27,28]. The study followed TriNetX’s data governance policies. Because the analyses used only aggregate, de-identified data that do not constitute human subjects research, this study was determined to be exempt from institutional review board review under the Common Rule (45 CFR §46.102(e)) and TriNetX’s data governance policies; the requirement for informed consent was therefore waived. This study followed the Strengthening the Reporting of Observational Studies in Epidemiology (STROBE) reporting guidelines [31].

### Study population

Adults aged 18–90 years with at least one clinical encounter between January 1, 2016, and December 31, 2022, were eligible. The loneliness-exposed cohort comprised patients with an ICD-10-CM diagnosis of loneliness and related social disconnection recorded in this period (primary analysis). Although loneliness, social isolation, and burn-out are conceptually distinct, they frequently co-occur, share overlapping neurobiological and psychosocial pathways, and are rarely differentiated in administrative coding; this composite operationalization follows prior EHR-based loneliness research. Burn-out (Z73.0) was retained within this composite because, in clinical risk modeling, it functions as a marker of chronic psychosocial and relational depletion that frequently coexists with, and is difficult to distinguish administratively from, acute loneliness and social disconnection; its inclusion reflects the way these states are jointly documented in electronic health records rather than an assertion of etiologic equivalence, and its contribution was directly interrogated in the prespecified sensitivity analysis that excluded it. The four codes were: problems of adjustment to life-cycle transitions (Z60.0), problems related to living alone (Z60.2), social exclusion and rejection (Z60.4), and burn-out (Z73.0). To assess robustness to this composite definition, a prespecified sensitivity analysis restricted the cohort to Z60.0 or Z60.2 only the two codes most directly capturing subjective loneliness yielding a matched cohort of 2,990 per arm (see Table S1). Eligibility also required a documented encounter (ambulatory, emergency, inpatient, home health, observation, or virtual) within 1 year preceding the first loneliness diagnosis. The comparator cohort comprised patients with at least one new-patient and one established-patient outpatient evaluation and management visit in the same period and prior healthcare activity, with no loneliness or burn-out diagnosis at any time. Patients with palliative care encounters (Z51.5, Z51.89) were excluded from both cohorts.

### Outcomes and follow-up

The primary outcome was all-cause mortality, ascertained from EHR mortality status fields. Three windows were prespecified: 1 year (days 1–365), 3 years (days 1–1,095), and 5 years (days 1–1,825) after the index event. Patients with the outcome before a window start were excluded from that analysis. The index event was the first qualifying loneliness diagnosis (loneliness cohort) or first qualifying outpatient visit pair (comparator cohort) within the study period.

### Covariates

Covariates were selected a priori based on established associations with both loneliness and mortality and their availability as structured ICD-10-CM fields within TriNetX [32,33]. Sociodemographic covariates included age at index (continuous), sex (male, female), and race and ethnicity (White, Black or African American, Hispanic or Latino, other or unknown), consistent with US federal standards.

Twelve comorbidity categories were operationalized using ICD-10-CM code ranges: ischemic heart disease (I20–I25); heart failure (I50); cerebrovascular disease (I60–I69); diabetes mellitus types 1 and 2 (E08–E13); chronic kidney disease (N18); chronic lower respiratory disease, including chronic obstructive pulmonary disease and asthma (J40–J4A); anxiety disorders (F41); mental and behavioral disorders due to psychoactive substance use (F10–F19); overweight and obesity (E66); personal history of nicotine dependence (Z87.891); neoplasms, encompassing all primary malignancies (C00–D49); and mood disorders, including depressive and bipolar spectrum conditions (F30–F39) [34]. Mood and anxiety disorders were included as covariates rather than excluded as mediators, consistent with estimating the independent association of loneliness with mortality beyond its known psychiatric comorbidity burden [35,36].

Before matching, the cohorts differed substantially across all covariates (SMDs, 0.059 to 0.580). After 1:1 PSM, all SMDs were less than 0.03 for demographic characteristics and less than 0.02 for comorbidity domains, confirming excellent balance. The matched sample comprised 28,844 patients per arm for the overall analysis.

### Propensity score matching

To minimize confounding, 1:1 nearest-neighbor propensity score matching (PSM) was performed on the TriNetX platform using age at index, sex, race and ethnicity, and the 12 comorbidity categories. Balance was assessed with standardized mean differences (SMDs); values less than 0.10 indicated adequate balance. For all subgroup analyses (sex, race and ethnicity, age group), PSM was performed separately within each stratum.

### Statistical analysis

For each window, cumulative mortality risk, risk difference (RD), risk ratio (RR), and odds ratio (OR) with 95% confidence intervals were calculated. Survival analyses used the Kaplan-Meier method with log-rank comparison. Hazard ratios (HRs) with 95% CIs were estimated using Cox proportional hazards models. Adherence to the proportional hazards’ assumption was evaluated via Schoenfeld residual–based testing; where violated (p > .05), HRs were reported as average hazard ratios, and log-rank and risk-based measures served as primary inference tools. Subgroup analyses were conducted by sex (female, male), race and ethnicity (White, Black or African American, Hispanic or Latino), and age group (working-age adults [18–64 years] and older adults [65–90 years]). Analyses used the TriNetX platform with a two-sided α = .05. No imputation was performed for missing data.

## Results

### Study population and propensity score matching

Before matching, the loneliness cohort (n = 28,945) and comparator cohort (n = 5,287,224) differed substantially across all covariates (Table 1). Patients with loneliness were older (mean age, 55.4 ± 24.2 vs 49.0 ± 20.2 years; standardized difference [SD], 0.285), more frequently female (62.9% vs 60.0%; SD, 0.059), and carried higher burdens of mood disorders (41.0% vs 15.9%; SD, 0.580) and anxiety disorders (34.9% vs 16.6%; SD, 0.426). After 1:1 matching, cohorts were balanced at 28,944 each; all post-match SDs were less than 0.03 (Table 1).

**Table 1:** Baseline characteristics of patients with and without a clinically documented loneliness diagnosis before and after propensity score matching.

Characteristic	Before matching Loneliness (n = 28 945)	Before matching No loneliness (n = 5 287 224)	Std. Diff.	After matching Loneliness (n = 28 944)	After matching No loneliness (n = 28 944)	Std. Diff.
**Demographics**						
Age at index, mean (SD), y	55.4 (24.2)	49.0 (20.2)	0.285	55.4 (24.2)	54.7 (23.3)	0.028
Female sex	18 195 (62.9)	3 171 059 (60.0)	0.059	18 194 (62.9)	18 018 (62.3)	0.013
Male sex	10 743 (37.1)	2 114 646 (40.0)	0.059	10 743 (37.1)	10 889 (37.6)	0.010
White race	19 713 (68.1)	3 819 709 (72.2)	0.091	19 713 (68.1)	19 813 (68.5)	0.007
Black or African American race	5412 (18.7)	723 003 (13.7)	0.137	5411 (18.7)	5352 (18.5)	0.005
Not Hispanic or Latino ethnicity	22 896 (79.1)	3 737 000 (70.7)	0.195	22 895 (79.1)	22 942 (79.3)	0.004
**Comorbidities**						
Ischemic heart disease	5428 (18.8)	460 184 (8.7)	0.295	5427 (18.8)	5432 (18.8)	<0.001
Heart failure	3695 (12.8)	205 379 (3.9)	0.326	3694 (12.8)	3645 (12.6)	0.005
Cerebrovascular disease	3561 (12.3)	259 935 (4.9)	0.266	3560 (12.3)	3518 (12.2)	0.004
Diabetes mellitus	6820 (23.6)	722 772 (13.7)	0.256	6819 (23.6)	6725 (23.2)	0.008
Chronic kidney disease	4041 (14.0)	279 602 (5.3)	0.297	4040 (14.0)	4036 (13.9)	<0.001
Chronic lower respiratory disease	7161 (24.7)	796 906 (15.1)	0.244	7160 (24.7)	7180 (24.8)	0.002
Overweight or obesity	7129 (24.6)	881 997 (16.7)	0.197	7128 (24.6)	6967 (24.1)	0.013
Neoplasms	8116 (28.0)	1 194 790 (22.6)	0.125	8116 (28.0)	7908 (27.3)	0.016
**Mental health diagnoses**						
Mood (affective) disorders	11 863 (41.0)	838 405 (15.9)	0.580	11 862 (41.0)	12 082 (41.7)	0.015
Anxiety disorders	10 089 (34.9)	879 874 (16.6)	0.426	10 088 (34.9)	10 305 (35.6)	0.016
Substance use disorders	7141 (24.7)	710 077 (13.4)	0.289	7140 (24.7)	7065 (24.4)	0.006
**Other**						
History of nicotine dependence	5426 (18.7)	502 015 (9.5)	0.268	5425 (18.7)	5272 (18.2)	0.014

**Abbreviations:** IQR, interquartile range; SD, standard deviation. Data are No. (%) unless otherwise indicated. **Cohorts:** Adults aged 18–90 years in the TriNetX US Collaborative Network (65 health care organizations; index period January 1, 2016, to December 31, 2022). The loneliness cohort comprised patients with a clinically documented loneliness diagnosis and a qualifying encounter in the prior year; the comparison cohort comprised patients with qualifying outpatient encounters and no loneliness diagnosis. Patients with palliative care codes were excluded. **Matching:** 1:1 propensity score matching used the listed characteristics as covariates. After matching, all standardized mean differences were less than 0.03, indicating excellent covariate balance.

### Overall cohort: All-cause mortality

Loneliness was significantly associated with all-cause mortality at every horizon (Table 2). At 1 year, 1280 deaths occurred in the loneliness cohort (4.4%) vs 866 in controls (3.0%; risk difference [RD], 1.4 percentage points [95% CI, 1.1–1.7]; hazard ratio [HR], 1.52 [95% CI, 1.40–1.66]; P < .001). At 3 years, cumulative mortality was 8.8% versus 6.9% (RD, 1.9 pp [95% CI, 1.5–2.3]; HR, 1.35 [95% CI, 1.27–1.43]; P < .001). At 5 years, 3270 deaths occurred (11.3%) vs 2800 in controls (9.7%; RD, 1.6 pp [95% CI, 1.1–2.1]; HR, 1.29 [95% CI, 1.22–1.35]; P < .001). Proportional hazards held at all three horizons.

**Table 2:** All-cause mortality at 1, 3, and 5 years among propensity score–matched adults with and without a loneliness diagnosis.

Follow-up window	Deaths, No. Loneliness	Deaths, No. No loneliness	Cumulative mortality, % (loneliness vs no loneliness)	Risk difference, percentage points (95% CI)	Risk ratio (95% CI)	Hazard ratio (95% CI)	Survival at end of window, % (gap, pp)
**1 year**	1280	866	4.4 vs 3.0	1.4 (1.1-1.7)	1.48 (1.36-1.61)	1.52 (1.40-1.66)	95.28 vs 96.85 (1.57)
**3 years**	2542	1993	8.8 vs 6.9	1.9 (1.5-2.3)	1.28 (1.21-1.35)	1.35 (1.27-1.43)	89.98 vs 92.37 (2.39)
**5 years**	3270	2800	11.3 vs 9.7	1.6 (1.1-2.1)	1.17 (1.11-1.23)	1.29 (1.22-1.35)	85.41 vs 88.23 (2.82)

**Abbreviations:** CI, confidence interval; pp, percentage points. **Cohorts:** Propensity score–matched adults with and without a clinically documented loneliness diagnosis (28 944 per group). **Measures:** Cumulative mortality is the proportion of deaths within each window; survival is the Kaplan-Meier estimate at the end of the window, and the gap is the loneliness vs no-loneliness difference. Risk differences and risk ratios derive from cumulative incidence; hazard ratios from Cox proportional hazards models. All risk differences, risk ratios, hazard ratios, and log-rank tests were significant at P < .001, and the proportional hazards assumption was met at all 3 horizons. Patients with the outcome before a given window were excluded from that window.

A coherent temporal pattern emerged: relative effects attenuated progressively (HR, 1.52 → 1.35 → 1.29), consistent with survivor selection, while absolute Kaplan-Meier survival gaps widened monotonically (1 year, 1.57 percentage points; 3 years, 2.39 pp; 5 years, 2.82 pp). The first year carried the greatest relative excess hazard, and the 5-year window the greatest absolute divergence.

### Subgroup analysis by sex

Sex-stratified analyses revealed a marked disparity (Table 3). Male patients with loneliness faced higher excess 1-year mortality (5.7% vs 3.2%; HR, 1.85 [95% CI, 1.63–2.12]) than female patients (3.7% vs 2.8%; HR, 1.35 [95% CI, 1.20–1.51]), with an absolute 1-year risk difference in males (2.5 pp [95% CI, 1.9–3.0]) nearly three times that in females (0.9 pp [95% CI, 0.5–1.3]). The disparity persisted through 5 years (male risk difference, 2.5 pp vs female, 1.2 pp; male HR, 1.40 [95% CI, 1.30–1.52] vs female HR, 1.22 [95% CI, 1.14–1.31]). Proportional hazards held in males at all horizons (all PH P < .001) but not in females at 3 years (P = .275) or 5 years (P = .091), indicating a decelerating hazard in women not seen in men.

**Table 3:** All-cause mortality by sex at 1, 3, and 5 years.

Subgroup	Deaths/No. Loneliness	Deaths/No. No loneliness	Mortality, % (L vs C)	Risk difference, percentage points (95% CI)	Risk ratio (95% CI)	Hazard ratio (95% CI)	P value log-rank; PH
**1-Year follow-up**							
**Female**	665/18 128	503/18 151	3.7 vs 2.8	0.9 (0.5–1.3)	1.32 (1.18–1.48)	**1.35 (1.20–1.51)**	<.001; < .001
**Male**	614/10 710	347/10 712	5.7 vs 3.2	2.5 (1.9–3.0)	1.77 (1.56–2.01)	**1.85 (1.63–2.12)**	<.001; < .001
**3-Year follow-up**							
**Female**	1406/18 128	1153/18 151	7.8 vs 6.4	1.4 (0.9–1.9)	1.22 (1.13–1.32)	**1.27 (1.17–1.37)** ^**a**^	<.001; .275
**Male**	1135/10 710	821/10 712	10.6 vs 7.7	2.9 (2.2–3.7)	1.38 (1.27–1.51)	**1.50 (1.37–1.64)**	<.001; < .001
**5-Year follow-up**							
**Female**	1870/18 128	1654/18 151	10.3 vs 9.1	1.2 (0.6–1.8)	1.13 (1.06–1.21)	**1.22 (1.14–1.31)** ^**a**^	<.001; .091
**Male**	1399/10 710	1133/10 712	13.1 vs 10.6	2.5 (1.6–3.3)	1.24 (1.15–1.33)	**1.40 (1.30–1.52)**	<.001; < .001

**Abbreviations:** C, no-loneliness control; CI, confidence interval; L, loneliness; PH, proportional hazards. **Matching:** 1:1 propensity score matching performed separately within each stratum on age, sex, race and ethnicity, and 12 comorbidity domains; TriNetX US Collaborative Network (65 health care organizations; 2016–2022). **Measures:** Risk difference is in percentage points; risk and hazard ratios with 95% CIs. The final column reports the Kaplan-Meier log-rank P value followed by the proportional hazards test P value. **Superscript a** indicates the proportional hazards assumption was not met (PH test P > .05); the hazard ratio at that horizon is an average rather than instantaneous estimate. **Shaded cells** denote estimates that did not reach statistical significance (95% CI crosses the null). Patients with the outcome before a given window were excluded from that window. **Sex-specific note:** The proportional hazards assumption was met in male patients at every horizon but violated in female patients at 3 and 5 years, consistent with a decelerating hazard trajectory.

### Subgroup analysis by race and ethnicity

Significant associations were observed in White and Black patients across all three horizons, and in Hispanic patients at 1 year (Table 4). White patients (n = 19 655 vs 19 677) showed estimates mirroring the overall cohort (1-year HR, 1.55; 5-year HR, 1.22), with proportional hazards satisfied throughout. Black patients (n = 5381 vs 5392) showed a distinctive intermediate-term excess: the 3-year risk difference (2.1 pp [95% CI, 1.1–3.0]) exceeded that of White patients (1.7 pp) and the overall cohort (1.9 pp), despite a smaller 1-year difference (0.9 pp); proportional hazards was not met at 3 and 5 years (PH P = .654 and .324), so those hazard ratios are average rather than instantaneous estimates. Hispanic patients (n = 2700 per arm) had the largest 1-year relative effect of any subgroup (RR, 2.33 [95% CI, 1.35–4.04]; average HR, 2.41 [95% CI, 1.39–4.18]), but associations were non-significant beyond 1 year (3-year log-rank P = .106; 5-year, P = .210), consistent with the Hispanic Paradox and limited power from low background mortality.

**Table 4:** All-cause mortality by race and ethnicity at 1, 3, and 5 years.

Subgroup	Deaths/No. Loneliness	Deaths/No. No loneliness	Mortality, % (L vs C)	Risk difference, percentage points (95% CI)	Risk ratio (95% CI)	Hazard ratio (95% CI)	P value log-rank; PH
**1-Year follow-up**							
**White**	955/19 655	636/19 677	4.9 vs 3.2	1.6 (1.2–2.0)	1.50 (1.36–1.66)	**1.55 (1.41–1.72)**	<.001; < .001
**Black or African American**	194/5381	145/5392	3.6 vs 2.7	0.9 (0.3–1.6)	1.34 (1.09–1.66)	**1.37 (1.10–1.70)**	.004; .011
**Hispanic or Latino**	42/2700	18/2700	1.6 vs 0.7	0.9 (0.3–1.4)	2.33 (1.35–4.04)	**2.41 (1.39–4.18)** ^**a**^	.001; .225
**3-Year follow-up**							
**White**	1862/19 655	1520/19 677	9.5 vs 7.7	1.7 (1.2–2.3)	1.23 (1.15–1.31)	**1.31 (1.22–1.40)**	<.001; < .001
**Black or African American**	420/5381	310/5392	7.8 vs 5.7	2.1 (1.1–3.0)	1.36 (1.18–1.56)	**1.42 (1.22–1.64)** ^**a**^	<.001; .654
**Hispanic or Latino**	71/2700	55/2700	2.6 vs 2.0	0.6 (−0.2–1.4)	1.29 (0.91–1.83)	**1.34 (0.94–1.90)** ^**a**^	.106; .010
**5-Year follow-up**							
**White**	2396/19 655	2173/19 677	12.2 vs 11.0	1.1 (0.5–1.8)	1.10 (1.05–1.17)	**1.22 (1.16–1.30)**	<.001; < .001
**Black or African American**	525/5381	436/5392	9.8 vs 8.1	1.7 (0.6–2.7)	1.21 (1.07–1.36)	**1.32 (1.16–1.50)** ^**a**^	<.001; .324
**Hispanic or Latino**	100/2700	87/2700	3.7 vs 3.2	0.5 (−0.5–1.5)	1.15 (0.87–1.52)	**1.20 (0.90–1.60)** ^**a**^	.210; .060

**Abbreviations:** C, no-loneliness control; CI, confidence interval; L, loneliness; PH, proportional hazards. **Matching:** 1:1 propensity score matching performed separately within each stratum on age, sex, race and ethnicity, and 12 comorbidity domains; TriNetX US Collaborative Network (65 health care organizations; 2016–2022). **Measures:** Risk difference is in percentage points; risk and hazard ratios with 95% CIs. The final column reports the Kaplan-Meier log-rank P value followed by the proportional hazards test P value. **Superscript a** indicates the proportional hazards assumption was not met (PH test P > .05); the hazard ratio at that horizon is an average rather than instantaneous estimate. **Shaded cells** denote estimates that did not reach statistical significance (95% CI crosses the null). Patients with the outcome before a given window were excluded from that window. **Hispanic subgroup note:** This was the smallest stratum (2700 per arm); 3- and 5-year estimates are exploratory, and low background mortality further constrains power at extended horizons.

### Subgroup analysis by age group

Working-age adults (18–64 years; n = 13 652 vs 13 659) showed significant excess mortality at all three horizons despite low event rates (1-year risk, 0.9% vs 0.6%; Table 5). Their hazard estimates were the most temporally stable of any subgroup (average HR range, 1.34–1.42), reflecting persistent, non-attenuating risk during peak productive years. Older adults (65–90 years; n = 15 193 vs 15 196) had the largest 1-year absolute risk difference of any stratum (2.0 pp [95% CI, 1.5–2.6]; HR, 1.42 [95% CI, 1.30–1.55]), identifying the immediate post-diagnosis period as the critical intervention window. At 5 years, risk-based measures were non-significant in older adults (RD, 0.8 pp [95% CI, −0.1 to 1.6]; RR, 1.04 [95% CI, 0.99–1.09]), while the hazard ratio remained highly significant (HR, 1.19 [95% CI, 1.13–1.25]; log-rank P < .001), reflecting compression of absolute differences by high background mortality.

**Table 5:** All-cause mortality by age group at 1, 3, and 5 years.

Subgroup	Deaths/No. Loneliness	Deaths/No. No loneliness	Mortality, % (L vs C)	Risk difference, percentage points (95% CI)	Risk ratio (95% CI)	Hazard ratio (95% CI)	P value log-rank; PH
**1-Year follow-up**							
**Working-age adults, 18–64 y**	118/13 652	84/13 659	0.9 vs 0.6	0.2 (0.0–0.5)	1.41 (1.06–1.86)	**1.42 (1.07–1.87)** ^**a**^	.014; .171
**Older adults, 65–90 y**	1162/15 193	854/15 196	7.6 vs 5.6	2.0 (1.5–2.6)	1.36 (1.25–1.48)	**1.42 (1.30–1.55)**	<.001; < .001
**3-Year follow-up**							
**Working-age adults, 18–64 y**	250/13 652	186/13 659	1.8 vs 1.4	0.5 (0.2–0.8)	1.35 (1.11–1.62)	**1.37 (1.13–1.66)** ^**a**^	.001; .417
**Older adults, 65–90 y**	2292/15 193	2003/15 196	15.1 vs 13.2	1.9 (1.1–2.7)	1.15 (1.08–1.21)	**1.24 (1.17–1.32)**	<.001; < .001
**5-Year follow-up**							
**Working-age adults, 18–64 y**	334/13 652	261/13 659	2.4 vs 1.9	0.5 (0.2–0.9)	1.28 (1.09–1.50)	**1.34 (1.14–1.57)** ^**a**^	<.001; .466
**Older adults, 65–90 y**	2936/15 193	2822/15 196	19.3 vs 18.6	0.8 (−0.1–1.6)	1.04 (0.99–1.09)	**1.19 (1.13–1.25)**	<.001; < .001

**Abbreviations:** C, no-loneliness control; CI, confidence interval; L, loneliness; PH, proportional hazards. **Matching:** 1:1 propensity score matching performed separately within each stratum on age, sex, race and ethnicity, and 12 comorbidity domains; TriNetX US Collaborative Network (65 health care organizations; 2016–2022). **Measures:** Risk difference is in percentage points; risk and hazard ratios with 95% CIs. The final column reports the Kaplan-Meier log-rank P value followed by the proportional hazards test P value. **Superscript a** indicates the proportional hazards assumption was not met (PH test P > .05); the hazard ratio at that horizon is an average rather than instantaneous estimate. **Shaded cells** denote estimates that did not reach statistical significance (95% CI crosses the null). Patients with the outcome before a given window were excluded from that window. **Older-adult note:** At 5 years the risk difference and risk ratio lost significance as high background mortality compressed the absolute gap, whereas the hazard ratio and log-rank test remained significant (HR, 1.19), confirming a persistent instantaneous excess hazard.

### Sensitivity analysis

In the prespecified sensitivity analysis restricting exposure to Z60.0 and Z60.2 only (n ≈ 2,990 per arm), the 1-year association was stronger than in the primary cohort. At 1 year, mortality was 5.1% vs 2.8% (RD, 2.3 percentage points [95% CI, 1.3–3.3]; RR, 1.82 [95% CI, 1.40–2.36]; HR, 2.21 [95% CI, 1.69–2.88]; P < .001). At 3 and 5 years, risk-based measures were non-significant (3-year RD, 0.5 pp [95% CI, −0.9 to 2.0], P = .451; 5-year RD, −0.5 pp [95% CI, −2.1 to 1.1], P = .564), but log-rank tests and hazard ratios remained significant at both horizons (3-year HR, 1.41 [95% CI, 1.19–1.69]; 5-year HR, 1.40 [95% CI, 1.20–1.63]; both log-rank P < .001), with proportional hazards violated at 5 years. The amplified 1-year hazard alongside attenuated risk-based significance is consistent with a more severely affected subpopulation in a smaller sample with reduced power and supports the robustness of the primary signal (Table S1).

## Discussion

In this propensity score–matched cohort study of 57,688 US adults from 65 health care organizations, clinically documented loneliness was independently associated with elevated all-cause mortality at 1, 3, and 5 years. Compared with matched controls, patients with loneliness faced a 52% higher hazard at 1 year (HR, 1.52 [95% CI, 1.40–1.66]) and a 29% higher hazard at 5 years (HR, 1.29 [95% CI, 1.22–1.35]), with absolute survival gaps widening progressively (1 year, 1.57 percentage points; 5 years, 2.82 pp). Subgroup analyses revealed substantial heterogeneity by sex, race and ethnicity, and age, with the largest relative effect in male patients at 1 year (HR, 1.85) and the largest 5-year absolute risk difference also in males (2.5 pp). These findings establish loneliness as a durable, clinically independent predictor of premature death across diverse US populations.

These results extend the existing evidence base in several respects. Prior meta-analyses suggest loneliness is associated with a 10–20% higher risk of all-cause mortality, with pooled HR around 1.14 [37]. Our estimates align with this range and, crucially, derive from clinically documented diagnoses rather than self-reported instruments, providing an actionable signal within health systems [38,39]. The attenuation of relative hazard from HR 1.52 at 1 year to 1.29 at 5 years is consistent with survivor selection, whereby high-risk individuals with the most severe isolation are lost to mortality early, attenuating the relative excess among survivors [40,41]. Importantly, the widening absolute survival gap despite attenuating relative hazard confirms that the cumulative mortality burden continues to grow over time, with direct implications for how loneliness should be framed as a public health priority [42].

The subgroup heterogeneity was clinically and epidemiologically important. Male patients bore a disproportionate burden, with 1-year HRs nearly 37% higher than female counterparts (1.85 vs 1.35), despite loneliness being diagnosed about 1.7 times more frequently in women. This likely reflects underdiagnosis in men, attenuated male help-seeking, and diminished access to informal support networks factors that may allow loneliness to progress to severe physiological dysregulation before clinical recognition [43–45]. Black patients showed a distinctive intermediate-term peak: the 3-year absolute survival gap (2.49 percentage points) exceeded that of White patients (2.28 pp) and the overall cohort (2.39 pp), consistent with delayed healthcare engagement and structural determinants that matching on comorbidities alone cannot neutralize [46,47]. Hispanic patients had the largest 1-year relative risk (RR, 2.33) but non-significant associations beyond 1 year, consistent with the Hispanic Paradox—lower background mortality driven by familismo and selective migration—combined with limited power from the smallest cohort (n = 2,700 per arm) [48,49]. Working-age adults (18–64 years) showed the most temporally stable excess hazard (HR range, 1.34–1.42), underscoring loneliness as a mortality driver during peak productive years, while older adults experienced the greatest first-year absolute impact (1-year RD, 2.0 percentage points), marking the immediate post-diagnosis period as the critical intervention window in geriatric care [5,50].

The mechanisms mediating loneliness-attributable mortality are likely multifactorial [51]. Chronic loneliness sustains hypothalamic-pituitary-adrenal hyperactivation, producing elevated glucocorticoids, heightened sympathetic tone, and a pro-inflammatory transcriptional profile marked by upregulated NF-κB signaling and impaired type I interferon responses [15,52,53]. These effects accelerate cardiovascular disease, impair immune surveillance, and may promote tumor progression consistent with the broad all-cause, rather than cause-specific, signal observed here [54,55]. These findings support the US Surgeon General’s recommendation to integrate loneliness screening into routine care and argue for time-sensitive outreach in the first year after a new diagnosis—the window of greatest absolute mortality excess across nearly every subgroup [4].

This study has several limitations. First, ICD-10-CM codes may undercount true loneliness, particularly in men and in settings with low awareness of Z-code documentation, potentially attenuating estimates [56]. Second, PSM balances measured covariates but cannot eliminate unmeasured confounding, including socioeconomic status, social network quality, and health literacy [57,58]. Third, the small Hispanic subgroup limits precision at extended horizons, and larger, culturally diverse cohorts are needed More broadly, several subgroups—particularly the Hispanic stratum and the smaller race- and age-specific cells—contained modest numbers of events, so the corresponding estimates should be regarded as exploratory and interpreted with caution rather than as definitive effect sizes [49,59]. Fourth, cause-specific mortality data were unavailable, precluding attribution of excess deaths to specific pathways. Fifth, proportional hazards violations in female, Black, and working-age subgroups at longer horizons required average hazard ratio interpretation; time-varying and restricted mean survival time models would further characterize these dynamics. Sixth, the composite exposure spans loneliness, social isolation, and burn-out (Z60.0, Z60.2, Z60.4, Z73.0), which are related but distinct; a prespecified sensitivity analysis restricted to Z60.0 and Z60.2 (Table S1) yielded a larger 1-year hazard ratio and significant hazard ratios at all horizons, supporting the robustness of the primary signal [60,61].

## Conclusions

In this propensity score–matched cohort study, clinically documented loneliness was independently associated with higher all-cause mortality in US adults at 1, 3, and 5 years, with the magnitude and time course differing substantially by sex, race and ethnicity, and age. These findings identify loneliness as a measurable, durable mortality risk marker in routine clinical data and support evaluating whether systematic screening and targeted intervention can reduce this risk.

## Supporting information

S1 File PLOSOne_Human_Subjects_Checklist_CSA_TriNetX_792026. (DOCX)

S1 Table Sensitivity Analysis: All-Cause Mortality at 1, 3, and 5 Years Among Adults with Clinically Documented Loneliness (ICD-10-CM Z60.0 and Z60.2 Only) vs Matched Controls. Abbreviations: CI, confidence interval; HR, hazard ratio; ICD-10-CM, International Classification of Diseases, Tenth Revision, Clinical Modification; No., number; PH, proportional hazards; RD, risk difference. Exposure definition: Restricted to two ICD-10-CM codes that most directly correspond to the subjective loneliness construct: Z60.0 (Problems of adjustment to life-cycle transitions) and Z60.2 (Problems related to living alone). Codes Z60.4 (Social exclusion and rejection) and Z73.0 (Burn-out), included in the primary analysis, were excluded from this sensitivity analysis cohort (n  =  2,990 per arm after 1:1 propensity score matching). Interpretation: At 1-year follow-up, loneliness defined by Z60.0 and Z60.2 alone was significantly associated with higher all-cause mortality (HR, 2.21 [95% CI, 1.69–2.88]; p  <  .001), with an absolute risk difference of 2.3 percentage points. At 3 and 5 years, risk-based measures (RD, RR, OR) were non-significant, though the Kaplan-Meier log-rank test and hazard ratio remained significant at both horizons, indicating a time-varying hazard structure. Compared with the primary analysis (28,844 per arm; HRs: 1.52, 1.35, 1.29), the sensitivity cohort shows a larger 1-year HR (2.21) but attenuated risk-based significance at extended horizons, likely reflecting the substantially smaller matched sample (n  =  2,990 vs 28,844) and reduced statistical power. The persistence of a significant hazard ratio across all three windows supports the robustness of the primary findings.
(DOCX)
