# Supplementary material for: Loneliness and all-cause mortality in the United States
Source: PLoS One. 2026 Sep 25;21(9):e0354773. doi: 10.1371/journal.pone.0354773 (PMC13614585; doi:10.1371/journal.pone.0354773)
Supplement: Supplementary file 2 [file pone.0354773.s002.docx]

**S1 Table.** Sensitivity Analysis: All-Cause Mortality at 1, 3, and 5 Years Among Adults with Clinically Documented Loneliness (ICD-10-CM Z60.0 and Z60.2 Only) vs Matched Controls

| **Outcome Measure** | **1-Year Follow-up** | | **3-Year Follow-up** | | **5-Year Follow-up** | |
| --- | --- | --- | --- | --- | --- | --- |
|  | **Loneliness (n=2,990)** | **No Loneliness (n=2,990)** | **Loneliness (n=2,954)** | **No Loneliness (n=2,954)** | **Loneliness (n=2,954)** | **No Loneliness (n=2,954)** |
| Patients with Outcome, No. (%) | 151 (5.1) | 83 (2.8) | 254 (8.6) | 238 (8.1) | 324 (11.0) | 338 (11.4) |
| Risk | 0.051 | 0.028 | 0.086 | 0.081 | 0.110 | 0.114 |
| Risk Difference (95% CI) | 0.023 (0.013, 0.033) | | 0.005 (−0.009, 0.020) | | −0.005 (−0.021, 0.011) | |
| Risk Difference p Value | < .001 | | .451 | | .564 | |
| Risk Ratio (95% CI) | 1.82 (1.40, 2.36) | | 1.07 (0.90, 1.26) | | 0.96 (0.83, 1.11) | |
| Odds Ratio (95% CI) | 1.86 (1.42, 2.45) | | 1.07 (0.89, 1.29) | | 0.95 (0.81, 1.12) | |
| Survival Probability at End of Window | 93.71% | 97.06% | 87.83% | 90.80% | 80.95% | 85.61% |
| Log-Rank χ² (df=1) | 35.22 | | 14.86 | | 18.79 | |
| Log-Rank p Value | < .001 | | < .001 | | < .001 | |
| Hazard Ratio (95% CI) | 2.205 (1.687, 2.882) | | 1.414 (1.185, 1.689) | | 1.401 (1.202, 1.633) | |
| Hazard Ratio p Value | < .001 | | < .001 | | .068 (PH violated) | |

**Abbreviations:** CI, confidence interval; HR, hazard ratio; ICD-10-CM, International Classification of Diseases, Tenth Revision, Clinical Modification; No., number; PH, proportional hazards; RD, risk difference.

**Exposure definition:** Restricted to two ICD-10-CM codes that most directly correspond to the subjective loneliness construct: Z60.0 (Problems of adjustment to life-cycle transitions) and Z60.2 (Problems related to living alone). Codes Z60.4 (Social exclusion and rejection) and Z73.0 (Burn-out), included in the primary analysis, were excluded from this sensitivity analysis cohort (n = 2,990 per arm after 1:1 propensity score matching).

**Interpretation:** At 1-year follow-up, loneliness defined by Z60.0 and Z60.2 alone was significantly associated with higher all-cause mortality (HR, 2.21 [95% CI, 1.69–2.88]; p < .001), with an absolute risk difference of 2.3 percentage points. At 3 and 5 years, risk-based measures (RD, RR, OR) were non-significant, though the Kaplan-Meier log-rank test and hazard ratio remained significant at both horizons, indicating a time-varying hazard structure. Compared with the primary analysis (28,844 per arm; HRs: 1.52, 1.35, 1.29), the sensitivity cohort shows a larger 1-year HR (2.21) but attenuated risk-based significance at extended horizons, likely reflecting the substantially smaller matched sample (n = 2,990 vs 28,844) and reduced statistical power. The persistence of a significant hazard ratio across all three windows supports the robustness of the primary findings.
